# AKR7A3 rs1738023 association with susceptibility to female hepatocellular carcinoma and its role in AFB1 metabolism and tumor

**DOI:** 10.1007/s12672-026-05757-9

**Published:** 2026-08-24

**Authors:** Jiakao Zhang, Yuan Liao, Ketuan Huang, Yongfei He, Meifeng Chen, Shutian Mo, Qiang Gao, Xinping Ye, Tao Peng, Chuangye Han

**Affiliations:** 1 Department of Hepatobiliary Surgery, The First Affiliated Hospital of Guangxi Medical University, Shuang-Yong Rd. 6, Nanning, 530021 Guangxi Zhuang Autonomous Region People’s Republic of China; 2 Guangxi Key Laboratory of Early Prevention and Treatment for Regional High Frequency Tumor, Nanning, Guangxi Zhuang Autonomous Region People’s Republic of China; 3 Guangxi Key Laboratory of Enhanced Recovery After Surgery for Gastrointestinal Cancer, Nanning, Guangxi Zhuang Autonomous Region People’s Republic of China

**Keywords:** Hepatocellular carcinoma, AKR7A3, Aflatoxin

## Abstract

**Background:**

Hepatocellular carcinoma (HCC) is one of the most common cancer worldwide. In this study, we performed a two-stage exome-chip association analysis and found that the aldo-keto reductase family7 member A3 (AKR7A3) rs1738023 may be a potential susceptibility locus for HCC in females. We aimed to explore its role and mechanism.

**Methods:**

The association between genotype and phenotype was analyzed through GWAS method. The expression of AKR7A3 in cancer tissue and blood analysis by qRT-PCR. The relationship of AKR7A3 and aflatoxin B1 (AFB1) was also analyzed. The effect of AKR7A3 on the biological behavior of HCC cell line was investigated on proliferation and invasion. The potential mechanism was analyzed by transcriptome analysis and western blot.

**Results:**

Through genome-wide association analysis (GWAS), AKR7A3 (rs1738023), KIF2C (rs4342887), and CYP3A5 (rs6977165 and rs4646450) were found to be associated with susceptibility to hepatocellular carcinoma (HCC) in women. Further expression quantitative trait loci (eQTL) analysis showed that only AKR7A3 (rs1738023) was significantly associated with gene expression. The expression of AKR7A3 was significantly lower in HCC than adjacent non-tumorous tissues (*P* < 0.001). The genotype of rs1738023 was significantly associated with AKR7A3 expression (*P* = 0.0085). Rs1738023[C] genotype had a low AKR7A3 expression level and limited detoxification ability of AFB1. Literature data showed that AKR7A3 is involved in the metabolism of aflatoxin B1 (AFB1). Functional experimental results showed that overexpression of AKR7A3 in the normal liver cell line HL-7702 could significantly reduce AFB1-induced ROS levels and DNA adduct formation, suggesting that it plays a protective role in AFB1 metabolic detoxification. Cell function test showed that overexpression of AKR7A3 inhibit the proliferation, migration and invasion of HCC cells, and block the cell cycle. Transcriptome sequencing and KEGG pathway enrichment analysis revealed that overexpression of AKR7A3 affected the PI3K signaling pathway and led to downregulation of HIF1A and its downstream VEGFA protein expression. The validation results were confirmed in HCC cell lines Huh-7 and SUN-387.

**Conclusion:**

Overexpression of AKR7A3 contributes to inhibition of HCC progression and reduction of aflatoxin toxicity. AKR7A3 may serve as a potential prognostic and therapeutic target for HCC patients, although further validation is needed.

**Supplementary Information:**

The online version contains supplementary material available at https://doi.org/10.1007/s12672-026-05757-9.

## Introduction

Primary liver cancer (PLC) is one of the most prevalent malignancies worldwide, among which hepatocellular carcinoma (HCC) accounts for approximately 75–85% of cases [1–3]. Nearly half of newly diagnosed HCC cases occur in China, and the incidence in Guangxi Zhuang Autonomous Region is substantially higher than the national average. This elevated burden has been largely attributed to the coexistence of chronic hepatitis B virus (HBV) infection and aflatoxin B1 (AFB1) exposure, two well-established risk factors that act synergistically in hepatocarcinogenesis [4–8]. The subtropical climate of Guangxi favors AFB1 contamination, while the prevalence of hepatitis B surface antigen (HBsAg) positivity remains high at approximately 7% [9]. Furthermore, the high frequency of the TP53 249Ser mutation, a molecular signature of AFB1 exposure, further supports the unique etiological background of this region [10–12].

HCC exhibits a pronounced sex disparity, with male incidence rates substantially exceeding those in females, particularly in HBV-related HCC [13]. Previous GWAS have identified several susceptibility loci associated with HCC risk, including variants located at 1p36.22 and 6p21.32 [14, 15]. However, most of these studies were conducted in mixed-sex populations. Consequently, genetic variants specifically associated with female susceptibility may be overlooked due to the predominance of male cases. Indeed, Zhang et al. demonstrated that rs17401966, a locus significantly associated with HCC risk in males, showed no significant association in females, highlighting the importance of sex-stratified genetic analyses [13, 15].

Nevertheless, female-specific susceptibility loci for HCC remain largely unexplored.

To address this knowledge gap, we performed a two-stage exome-chip association study in women from Guangxi, a region characterized by high HBV and AFB1 exposure, to identify genetic variants associated with susceptibility to HBV-related HCC. Functional experiments were subsequently conducted to evaluate the biological role of the candidate gene, while transcriptome sequencing was employed to explore the underlying molecular mechanisms. In addition, aflatoxin metabolism assays were performed to investigate the potential involvement of the identified susceptibility gene in AFB1 detoxification.

## Materials and methods

### Patients and specimens

The tissues of 107 female patients with HBV-related primary hepatocellular carcinoma treated in hepatobiliary surgery from January 2001 to November 2013 were collected as the case group. DNA was extracted from the blood of 311 female HBV carriers for follow-up GWAS analysis. The cancer and adjacent non-cancerous samples of 81 patients in hepatobiliary surgery from January 2013 to November 2018 were collected for follow-up qRT-PCR detection to verify the expression of target gene AKR7A3. All patients underwent hepatectomy without any treatment before surgery and were pathologically confirmed as HCC. Samples were immediately stored at -80℃.

### GWAS(genome-wide association study)

This study was a two-stage exome-chip association study. In the discovery stage, we used the Illumina HumanExome BeadChip-12-1_A (Illumina, USA), which contains approximately 242,901 SNPs enriched for exonic functional variants. Quality control (QC) thresholds included: sample call rate > 95%, SNP call rate > 95%, minor allele frequency (MAF) ≥ 0.05, Hardy-Weinberg equilibrium (HWE) *P* ≥ 1 × 10⁻⁶ in controls, and identity-by-descent (IBD) ≤ 0.1875. Principal component analysis (PCA) using EIGENSOFT showed a genomic inflation factor λ = 1.008, indicating no significant population stratification. After QC, 53 cases and 129 controls with 25,208 SNPs entered association analysis. Association was tested using EPACTS 3.2.6 (Single Variant Test, logistic score test) with adjustment for age, smoking, and drinking. Due to the small sample size, we selected the top 30 SNPs (lowest P values) for replication. In the replication stage, candidate SNPs were genotyped using the MassARRAY iPLEX Gold system in an independent cohort (53 cases, 180 controls) with the same QC criteria. Detailed procedures are provided in the Supplementary Materials (Sect.  1.3–1.5).

### Cell culture and lentivirus infection

Three hepatoma cell lines (Huh7, HepG2, SUN-387) were purchased from the Shanghai Cell Bank of the Chinese Academy of Sciences, and the human immortalized hepatocyte line HL-7702 was preserved by our group. We used Dulbecco’s Modified Eagle Medium (DMEM, BI, Israel), fetal bovine serum (FBS, Gibco, BI, Israel) and 1% penicillin-streptomycin solution (Gibco, CA, USA) to culture 4 cell lines in a 37 ℃ humidity incubator containing 5% CO2. We used lentivirus to establish 4 stable AKR7A3 over-expression cell lines, which was purchased from Genecopoeia company(USA).

### AKR7A3 expression verification and survival analysis

AKR7A3 expression was verified using the GSE14520 from the Gene Expression Omnibus (GEO) dataset [16, 17]. The relationship between AKR7A3 expression and prognosis were evaluated according to Kaplan-Meier Plotter (https://kmplot.com/) [18].

### Quantitative RT-PCR (qRT-PCR)

TRIzol reagent purchased from Solarbio (China) and then we extracted total RNA and transcribed into cDNA. Then, we use PCR Master Kit (QIAGEN, Germany) for qRT-PCR. The sequence was showed in Table S1. Follow the instructions for specific operation steps. The 2-∆∆Ct method and the original Ct value were used to measure the RNA expression levels [19].

### Western blotting

RIPA cleavage buffer (Beyotime, China) was used to lyse all cells and then extract total proteins. We used the BCA method to verify the concentration of the protein. The protein was separated by SDS-PAGE gels and transferred to polyvinylidene fluoride (PVDF) membrane. We washed PVDF membrane with PBST for 3 times. The PVDF membrane was incubated with primary antibody at 4℃ overnight. Antibody incubation with PVDF after washing at 25℃. All the antibodies used as follows: AKR7A3 Polyclonal antibody (13209-1-AP; Proteintech, USA); HRP-conjugated GAPDH (HRP-60004; Proteintech, USA); VEGFA Polyclonal antibody (19003-1-AP; Proteintech, USA); HIF-1α Polyclonal antibody (20960-1-AP; Proteintech, USA). The results were analyzed by ImageJ software.

### Cell proliferation, migration and invasion tests

All cell lines were separated into several groups and inoculated on 96-well plate at the density of 5 × 10^3^ cells/mL. Cell count Kit-8 (CCK-8 Kit; Dojindo, Japan) was used by the manufacturer’s instructions. We took measures of the optical density (OD) under 450 nm after 24, 48 and 72 h, respectively. Transwell was used to detect the invasion and metastasis. All the cell density of different groups was adjusted to 2 × 10^5^ cells/mL, 100 µL cell suspension were added to the chamber with or without Matrigel (Corning, USA) and cells were cultured for 48 h. And cells fixed with paraformaldehyde for 0.5 h, rinsed three times, and then rinsed with 1% crystal violet staining for 30 min. They were observed and photographed under a 5-field microscope.

### Wound healing test

Three kinds of cells and their corresponding overexpressed cells were digested. According to the grouping, 2 × 10^5^ cells per hole were placed in six-hole plate and incubated in 37℃ and 5%CO_2_ incubator until the cell density grew to 100%. Scratches were made in the six-hole plate with a 10-microliter liquid gun head and washed with PBS for three times. Photographs were taken at 0, 24, and 48 h. The blank area was calculated by Image J software.

### Flow cytometry test

The digested cells were divided into two groups: all cells per well were placed in a six-hole plate and incubated in a 5% CO_2_ incubator at 37℃ for 24 h. The cells were prepared with apoptosis and cycle detection kit (C1052, Beyotime, China), and then the distribution of cells in each cycle was analyzed by flow cytometry (BD Accuri C6 Plus; BD Biosciences, USA).

### RNA-sequencing and enrichment analysis

The second-generation sequencing of Huh-7 cells in the negative control (NC) group and the stable AKR7A3 over-expression group was completed by BGI (China). And we further analyzed the sequencing literature on the BGISEQ-500 platform. Then, DESeq2 was used for differential expression analysis, a | log Fold Change | > 2 and adjusted P values < 0.05 is regarded as different expressed genes (DEGs). In order to further explore its potential mechanism changes, the differential genes were analyzed by KEGG and GO enrichment analysis.

### Effects of AKR7A3 on metabolites of AFB1 phase 1 metabolites

AFB1 was purchased from Sheng gong Company, and liver micro particles from adult male SD rats were purchased from Qi’s Biotechnology Company. AFB1 (molecular weight 312.27) is dissolved in 3.21 ml DMSO, the concentration of 1mM AFB1 mother liquor is obtained. The PBS/DMEM complete medium mixture of AFB1 (final concentration of 1µM, 2µM, 5µM, 10µM, 100µM) + NADPH (final concentration of 2mM) + adult male SD rat liver particles (final concentration of 1.5 mg/mL) was incubated at 37 ℃ for 30 min, then 10 min was centrifuged with 4000 rpm and the supernatant was obtained.

A stable overexpressed HL-7702 cell line was constructed using lentivirus. Detection of AFB1-DNA adducts: drop 100uL complete medium into 12-well plate, spread cell climbing tablets, take HL-7702 cells in logarithmic growth phase (density 70%-80%), set over-expression group and control group, intervention completed AFB1-DNA determination, specific methods refer to ReginaM and Santella methods [1]. Three independent biological replicates were performed, each with triplicate technical replicates. The exposure duration was 24 h based on preliminary time-course experiments. AFB1-DNA adducts were detected by immunohistochemistry following Santella et al. (1993) [20], which is a semi-quantitative method.

The cells were digested and put into 1 ml medium containing 1 × 10^6^ cells in each well of 6-well plate and cultured in 5%CO_2_ incubator at 37℃ for 24 h. According to the group, DMEM complete culture medium containing different concentrations of supernatant was added to the cell plate and incubated for 24 h, and then treated according to the reactive oxygen content determination kit (S0033S, Beyotime, China). The fluorescence intensity was calculated and analyzed by Image J software.

### Statistical analyses

SPSS (version 22.0) was used in this study. Statistical charts were created using GraphPad Prism (version 8.0.1) and statistical differences between groups were evaluated according to the χ^2^ test or the rank sum test. Kaplan-Meier method was used to calculate the survival rates, and the log-rank test was performed to evaluate the difference of survival rates. The student’s t test was used to compare the two groups. All data are presented as mean ± SEM, with at least three independent biological replicates.

## Results

### GWAS study results

**Fig. 1:**
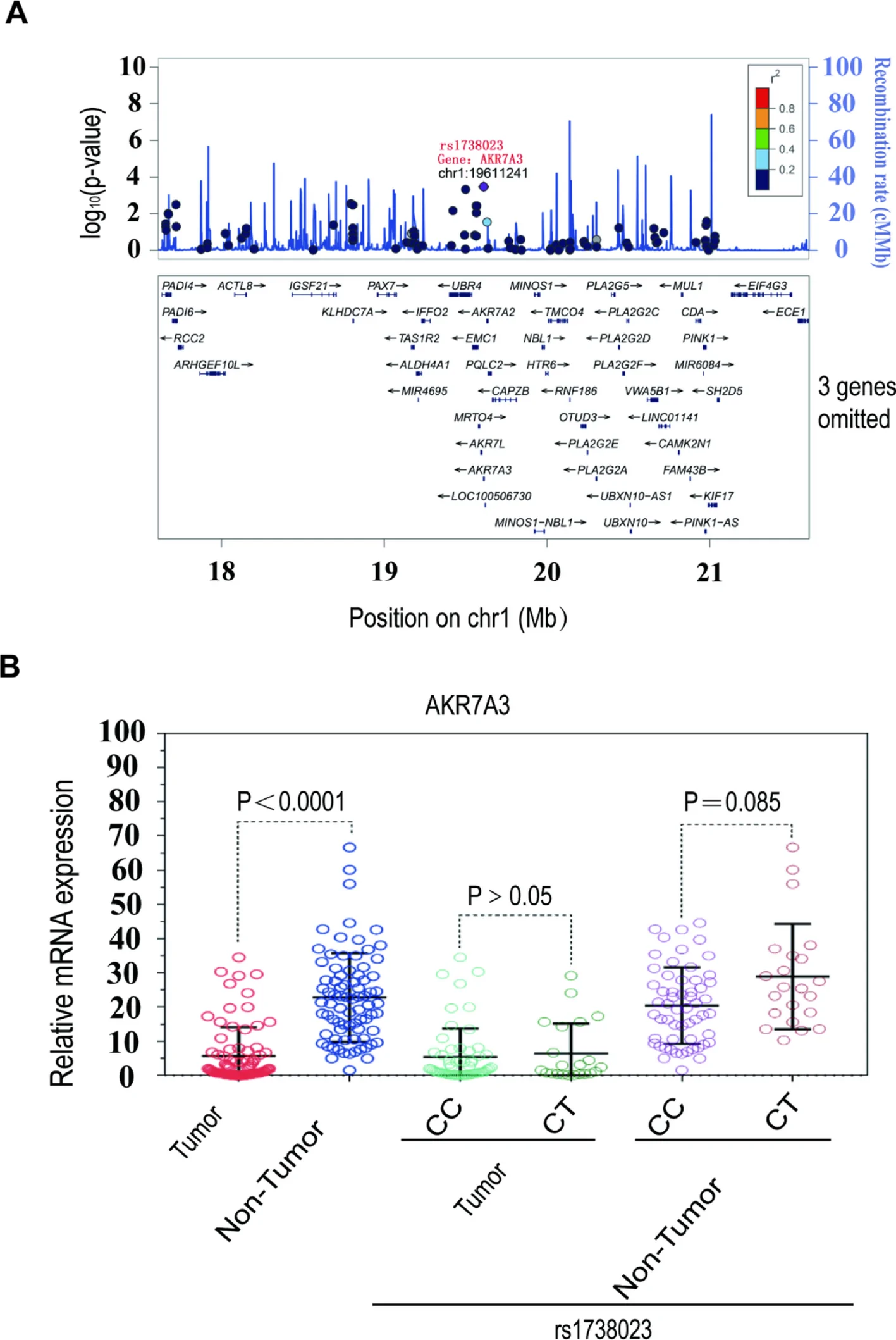
**A** Manhattan plots for association analysis. Results from Single Variant Test (−log10 P values) were plotted against genomic position (GRCh37/hg19). **B** Candidate genes and loci analyzed eQTL; light and dark green circles represent CC/CT genotype expression in cancer tissues; purple and orange circles represent CC/CT genotype expression in adjacent non‑tumorous tissues

Baseline characteristics of the GWAS stage population (53 cases, 129 controls) were as follows: mean age was 49.5 ± 11.5 years for cases and 48.8 ± 8.3 years for controls (*P* = 0.655); one smoker in the case group and two smokers in the control group (*P* = 0.291); two drinkers in the case group and two drinkers in the control group (*P* = 0.084). No significant differences were observed between the two groups for these variables, which were subsequently adjusted for in the multivariable logistic regression analysis.Before quality control, our study total included 107 case group and 311 cases control group, then genotyping of SNP loci in exons of the whole genome. Subsequently, we found that there were no significant outlier samples after principal component analysis (PCA) of quality control standard process (Fig S1A). Through homologous relationship identification, it was found that the identity-by-descent (IBD) of 4 pairs of samples was greater than 0.1875 (Fig S1B). After the standard process of quality control, half of the 4 pairs of samples were removed due to genetic relationship, that is, 2 cases in the case group and 2 cases in the control group. After the quality control procedure, a total of 105 case group and 309 cases control group were included in the clinical baseline data of the patients included in GWAS 434analysis, 25,208 SNP loci were included in association analysis, and the overall genotyping rate was 99.17%. The results of population stratification show that the gene expansion coefficient of the whole population is 1.008, indicating that there is no population stratification or stratification is not significant(Fig S1C). The exon SNP locus typing data of the whole genome after the quality control program, through the association analysis of EPACTS 3.2.6 software package, we get the P value of each locus, then we use R software to draw Manhattan map, and identify significant loci and genes for tagging in subsequent repeated experiments༈Fig. 1A༉. After correcting the exposure factors such as age, smoking and drinking, there was still a significant difference in the frequency of rs1738023 allele distribution between the two groups༈Table S2༉. To address sparse data (TT genotype absent in cases), we re‑analyzed the association using Firth’s penalized logistic regression, which confirmed that the C allele is the risk allele (OR = 2.45, 95% CI: 1.52–3.95, *P* = 0.0003). The eQTL(expression quantitative trait locus) analysis results shows that there was a significant correlation between the genotype of rs1738023 site and the mRNA expression in adjacent non‑tumorous tissues༈*P* = 0.0085༉. The expression of AKR7A3 was higher in rs1738023 CT genotype than that of CC genotype༈Fig. 1B༉.

### Verification and prognostic analysis

**Fig. 2:**
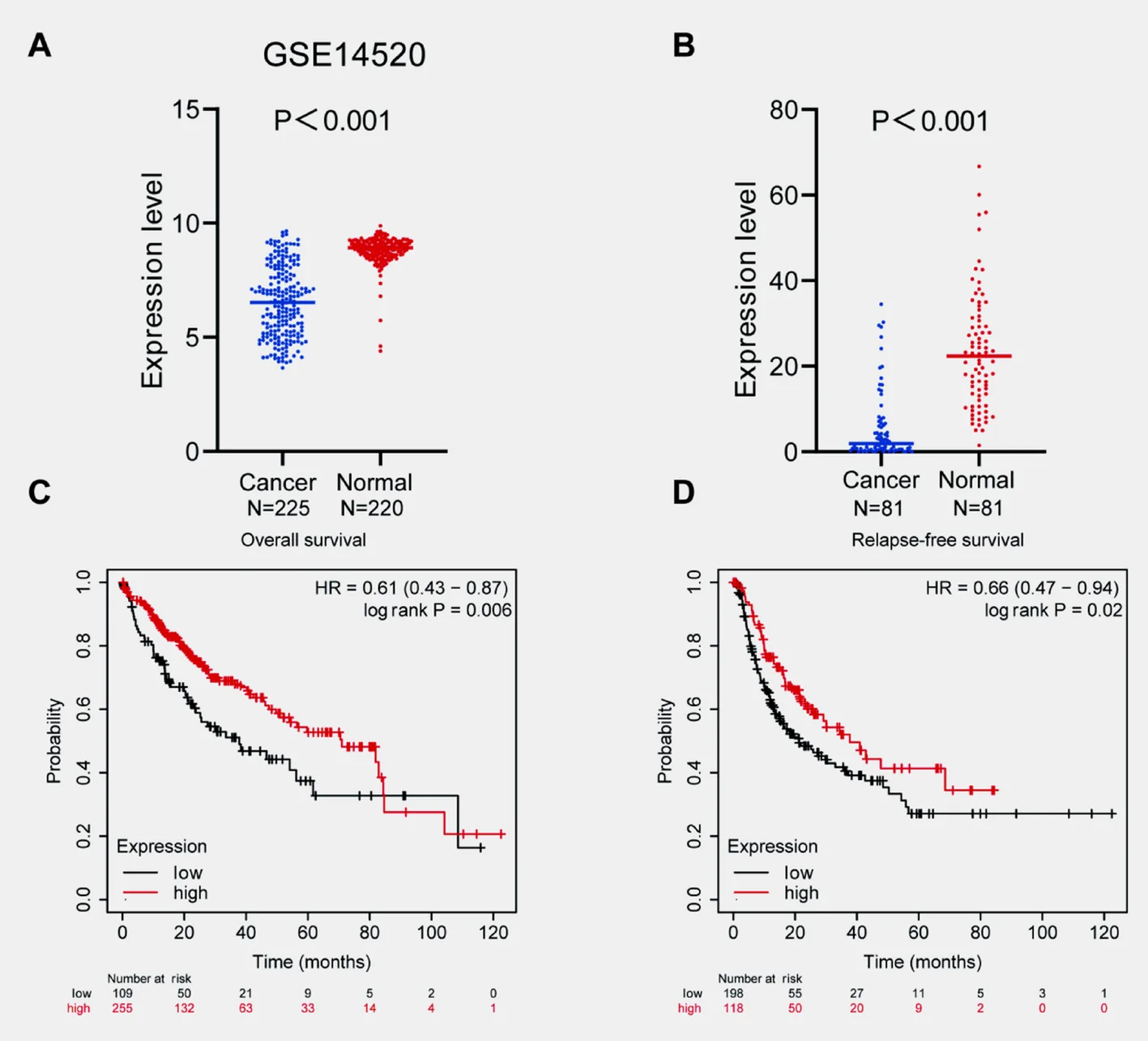
**A** Level of AKR7A3 expression in GSE14520 (GEO).**B** mRNA expression of AKR7A3 in 81 paired HCC and adjacent non-cancerous tissues based on real-time polymerase chain reaction analysis. **C** Overall survival curve. **D** Relapse-free survival curve

We further verified the expression of AKR7A3 inliver cancer and adjacent non-tumorous tissues. The expression level of AKR7A3 in HCC tissues was significantly decreased in GSE14520(Fig. 2A), At the same time, PCR was used to explore AKR7A3 expression in 81 pairs of HCC cancer and adjacent non-tumorous tissue samples, and the same results were observed (Fig. 2B). Survival analysis according to the Kaplan-Meier Plotter websites found that high AKR7A3 expression represent better overall survival and relapse-free survival. *P* = 0.006; *P* = 0.02. Fig. 2C and D.

### Effects of AKR7A3 gene on metabolites of AFB1 Phase 1 Metabolites

**Fig. 3:**
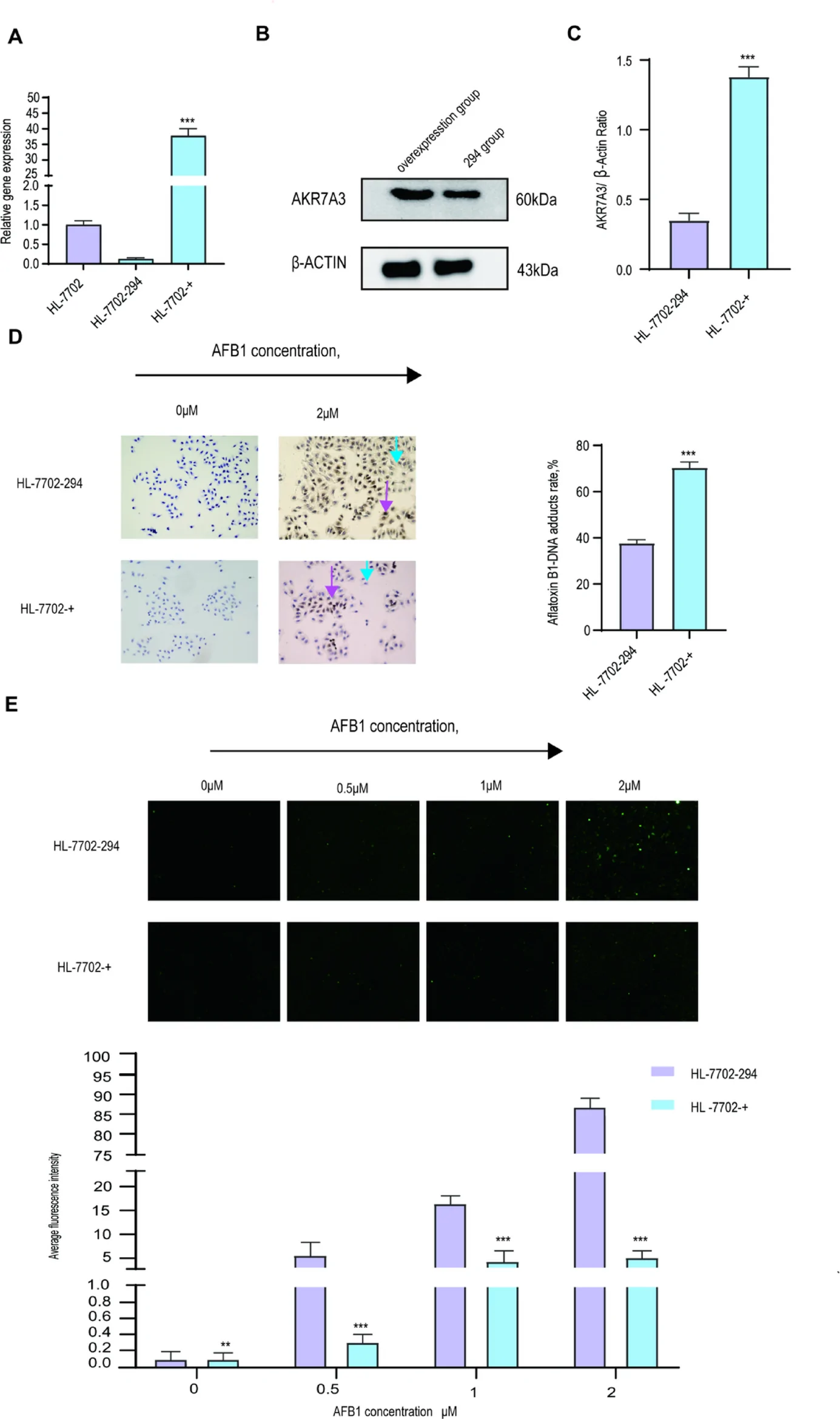
**A** Real-time polymerase chain reaction results for AKR7A3 expression in HL-7702 cell lines **B**,** C** Western blot results for AKR7A3 expression in HL-7702 cell lines and statistical analysis. **D** AFB1 adduct determination in HL-7702 cell lines; Green arrows represent normal 7702 cells and red arrows represent AFB1-DNA adducts **E** ROS detection in HL-7702 cell lines and statistical analysis. *,P＜0.05; **,P＜0.01; ***, P＜0.001; AKR7A3, aflatoxin reductase AFAR; CON294, Blank plasmid group; +, overexpression group

AKR7A3 over-expression HL-7702 cell line was constructed, and the over-expression efficiency of AKR7A3 was detected by qRT-PCR and western blot. AKR7A3 could be well overexpressed in HL-7702 cell line (Fig. 3A-C).

AFB1-DNA adduct determination and ROS detection results were shown in (Fig. 3D and E). AKR7A3 gene overexpression significantly reduced AFB1-DNA adduct formation and ROS levels in hepatocytes.

### Evaluation of AKR7A3 over-expression efficiency

**Fig. 4:**
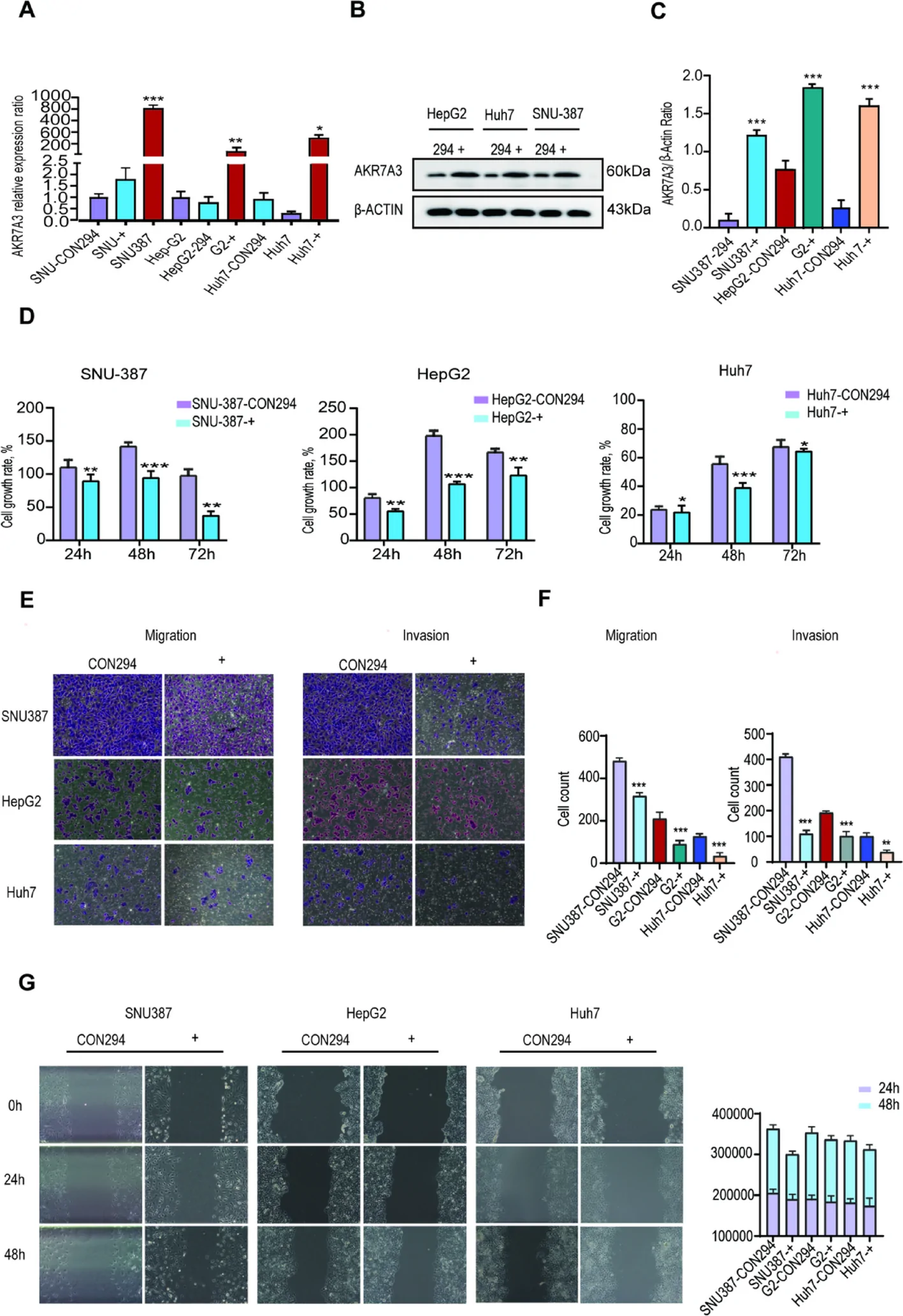
**A** Real-time polymerase chain reaction results for AKR7A3 expression in SUN387, Hep-G2 and Huh7 cell lines **B**,** C** Western blot results for AKR7A3 expression in SUN387, Hep-G2 and Huh7 cell lines and statistical analysis. **D** Cell growth rate for SUN-387, HepG2 and Huh7 cell lines based on the Cell Counting Kit-8 assay. **E**,** F** Representative images and statistical analysis of transwell migration and invasion assays in different groups and cell lines.*,*P*<0.05; **,*P*<0.01; ***, *P*<0.001; AKR7A3, aflatoxin reductase AFAR; CON294, Blank plasmid group; +, overexpression group. **G** Representative images and statistical analysis of wound-healing assay in different groups and cell lines. Scale bar, 100 μm

Western blot and qRT-PCR were used to detect the effect of AKR7A3 overexpression in the three cell lines. AKR7A3 was well over-expressed in all three cell lines (Fig. 4A-D).

### Overexpression of AKR7A3 inhibits proliferation, migration and invasion of HCC cell lines

The results of CCK-8 detection showed that the proliferation ability of HCC cells could be reduced by up-regulation the expression level of AKR7A3. Up-regulation expression of AKR7A3 can significantly reduce the proliferative ability (Fig. 4E-G). Transwell experiments showed that AKR7A3 over-expression could inhibit the invasion and metastasis of HCC cells(Fig. 4E and F). Similar results were obtained in wound healing trials, where up-regulation of AKR7A3 inhibited the migration activity of HCC cells, but the difference in healing area between the two groups was not statistically significant༈Fig. 4G༉.

### Overexpression of the AKR7A3 gene can prolong the cell cycle

**Fig. 5:**
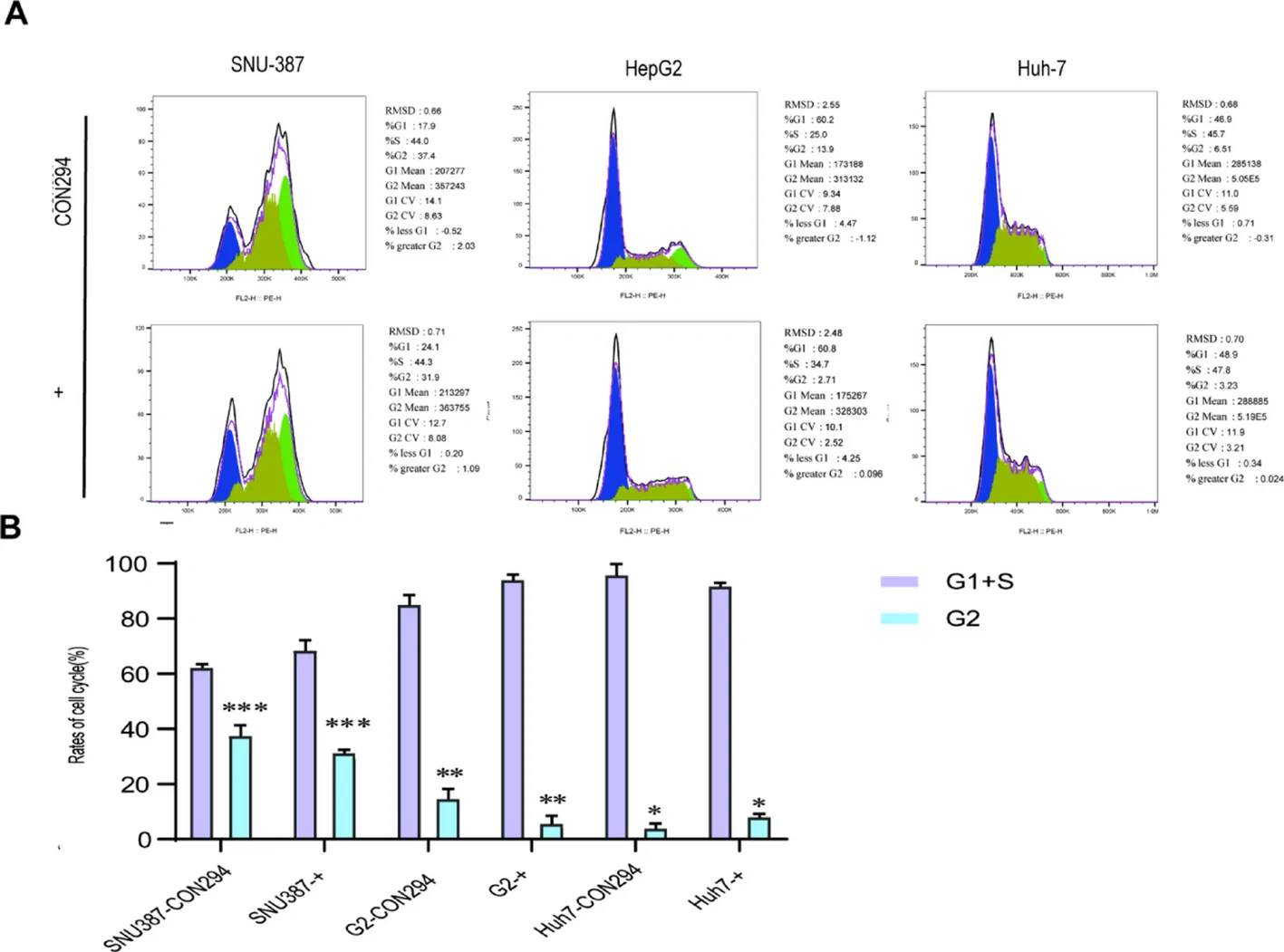
**A**,** B** Cell cycle distribution in each group calculated by flow cytometry assay and statistical analysis in different groups and cell lines. CON294, Blank plasmid group; +, AKR7A3 overexpression group. *,P＜0.05; **,P＜0.01; ***, P＜0.001; AKR7A3, aflatoxin reductase AFAR; CON294, Blank plasmid group; OD, optical density; +, overexpression group

Overexpression of the AKR7A3 gene increased the proportion of cells in G1/S phase and changed the cell cycle (Fig. 5A and B).

### Transcriptome sequence analysis

**Fig. 6:**
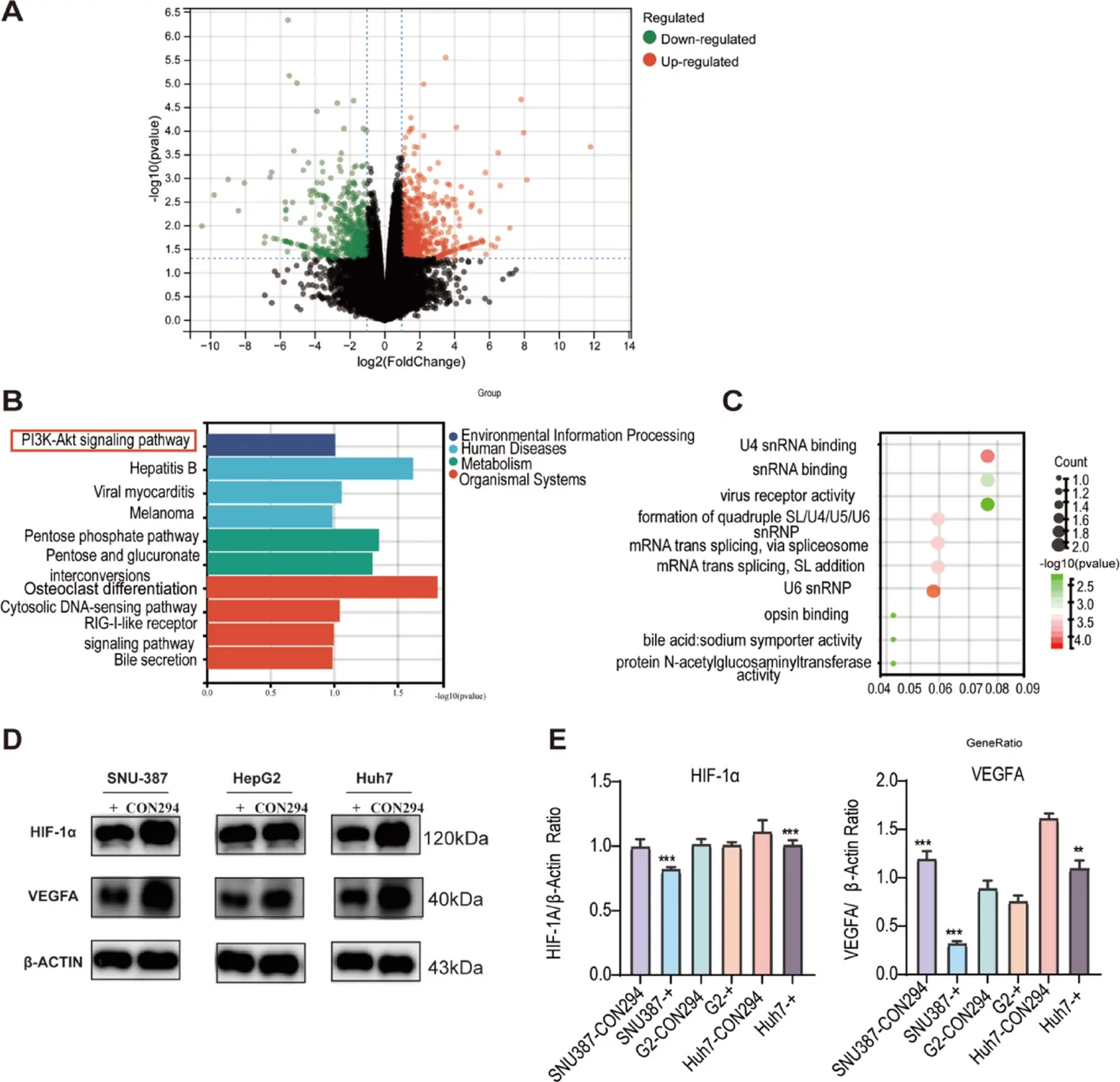
**A** Volcano map for differential gene (Huh7 vs. Huh7-+)**B** Top 20 GO enrichment based on differentially expressed mRNA from small to large according to P value. **C** Top 20 enrichment KEGG pathways based on differentially expressed mRNA from small to large according to P value. **D**,** E** Western blot results for HIF‑1α/VEGFA signaling pathway protein expression in SNU‑387, HepG2 and Huh7 cell lines and statistical analysis. CON294, blank plasmid group; +, AKR7A3 overexpression group

To further explore the potential mechanism of AKR7A3 in inhibiting HCC progression, we performed transcriptome sequencing on the AKR7A3 overexpression group and the control group. Differential analysis and enrichment analysis, including KEGG and GO, were performed. Differential expression analysis showed that 1119 mRNA transcripts (588 upregulated and 531 downregulated) were significantly different between the treatment group and the control group (Fig. 6A). Cluster analysis was performed on the differentially expressed mRNA profiles to identify different gene expression patterns. KEGG pathway enrichment analysis showed that the significantly affected genes may have an impact on pathways such as the PI3K signaling pathway and the pentose phosphate pathway (Fig. 6B). GO enrichment analysis of the differentially expressed mRNAs highlighted their involvement in metabolic processes, cellular components, and cellular processes (Fig. 6C).

### Pathway verification

Western blot was used to detect HIF-1α and VEGFA protein expression (Fig. 6D-G). Up‑regulation of AKR7A3 significantly reduced HIF‑1α and VEGFA protein expression in Huh7 and SNU‑387 cell lines. However, no significant difference in HIF‑1α expression was observed in HepG2 cells, which may be because HepG2 is a hepatoblastoma‑derived cell line, and its pathogenesis may differ from that of HCC.

## Discussion

The incidence of HCC was higher in males. According to the Cancer Statistics, for every 2.8 men who develop HCC, there is 1 woman who develops the disease, which can be as high as 7:1 in HBV-related HCC [21–23]. This gives us an important hint that HCC, as a tumor with a huge gender difference in incidence, should be studied separately between male and female populations when we study the genetic factors related to the pathogenesis, otherwise the genetic factors will be obscured by gender factors. With the deepening of human understanding of cancer, people gradually realize that the prevention of malignant tumor is the most effective treatment. Although the diagnosis and treatment of HCC has been greatly improved in recent years, the early diagnosis of HCC is still challenging, resulting in many patients being diagnosed in the late stage [24, 25]. In the face of the special environment of high HBV/AFB1 exposure in Guangxi, primary and secondary prevention of HCC in a large population is not only a medical issue, but also an economic one, so it is particularly important to identify vulnerable individuals among the high-risk groups for HCC. In a previous study by our group, we attempted to identify high-risk regions for HCC by investigating climate factors in the production of AFB1 in Guangxi, thus identifying high-risk groups for HCC. However, we did not achieve the desired result. Therefore, through the GWAS research strategy, we performed association analysis on a population of women with high HBV/AFB1 exposure backgrounds in an attempt to capture potentially susceptible gene loci, providing theoretical and preliminary experimental basis for the development of an early warning model of HBV-associated HCC in women.

To avoid certain key objectives being obscured by gender issues, this study was based on a female population with a high HBV/AFB1 exposure background. GWAS analysis of female HCC susceptibility showed that the expression of AKR7A3, CYP3A5, and KIF2C genes in liver cancer tissues and adjacent tissues and their corresponding genotypes at rs1738023, rs4342887, rs6977165, and rs4646450 sites were associated with female susceptibility. As shown in the Fig. 1, the genotype of the rs1738023 site was significantly associated with the mRNA expression of the AKR7A3 gene in adjacent tissues (*P* = 0.0085), and the expression of the CT genotype was higher than that of the CC genotype, but there was no significant difference in cancer tissues. We identified rs1738023 (located in AKR7A3 gene).To functionally analyze why the above three sites affect the susceptibility of female HCC Subsequently, we conducted eQTL analysis on the above loci and genes, and the results showed that rs1738023 genotype was significantly associated with AKR7A3 gene mRNA expression, The rs1738023 [C/T, Asn > Asp] locus located in the exon of AKR7A3 gene in the p36.13 region of chromosome 1 was significantly associated with HBV related HCC in women, and rs1738023 [C] was a risk genotype; the expression of AKR7A3 mRNA and protein in rs1738023 [C] genotype was significantly lower than that in rs1738023 [T] genotype; The expression of the AKR7A3 gene in HCC was significantly lower than in the corresponding neighboring tissue. AKR7A3 (aflatoxin reductase AFAR) is located on human chromosome 1p36 and is a member of the human AKR protein family. One of its important physiological functions is to reduce aflatoxin B1 dihydroate and reduce the toxicity of aflatoxin [8, 26]. Aflatoxin is an important pathogen in liver cancer, with 4.6–28.2% of global HCC cases attributed to AFB1 [27]. Considering the characteristics of high AFB1 exposure in Guangxi, we identified AKR7A3 as the target gene for subsequent experiments.

Through the expression of AKR7A3 and the detection of ROS and AFB1 adducts in HL-7702 cells after AFB1 treatment, it was demonstrated that overexpression of AKR7A3 significantly reduces ROS levels and AFB1-DNA adduct formation, further confirming the detoxifying effect of AKR7A3 on AFB1. Using liver tissue from F344/N rats exposed to AFB1 for 90 days, Merrick et al. [28]. found that a 14-gene combination, including the AKR7A3 gene, could serve as an important toxicological tool to assess key molecular events associated with chemical exposure. This suggests that AKR7A3 may play an important role in AFB1-induced hepatotoxicity and genotoxicity. In a study on β-naphthoflavone enhanced oxidative stress response and induced precancerous lesions in diethylnitrosamine-induced rat hepatocarcinogenesis model, the increased expression of GSTM1, GPX2, AKR7A3 and YC2 (as well as Nqo1) was detected and involved in the liver anti-oxidative stress protective effect [29]. It has been shown that in rabbits treated with prolonged/higher doses of oxfendazole, it increases the gene expression of not only phase I (CYP1A1 and CYP1A2) but also phase II (NQO1, GPX2, YC2 and AKR7A3) drug metabolism enzymes, and the results of this study suggest that, oxfendazole triggers an adaptive response against oxidative stress in the liver and suggests that an imbalance in REDOX status may be one of the factors triggering the initial step of oxfendazole induced nongenotoxic liver carcinogenesis in rats [30].

There is also direct evidence that overexpression of AKR7A3 gene in cell lines can effectively protect hepatocytes from AFB1 induced cell damage. In their study, compared with the control cell line, the half lethal dose of AFB1 increased from 30 μm/L to 300 μm/L in the cell line expressing AKR7A3 [26, 31], these results provide the first direct evidence for the role of human AKR7A3 in resisting AFB1 induced cytotoxicity and protein adduct formation. This is also consistent with our research results. These findings provide a functional explanation for how AKR7A3 (rs1738023) may affect the susceptibility of female liver cancer in a population with high AFB1/HBV co‑exposure (referring to regional background, not confirmed individual exposure).

To further verify whether this difference in gene expression holds for the entire HCC population, we performed qRT-PCR detection on clinical samples and found a significant reduction in the expression of AKR7A3 in tumor tissue. We validate our findings using public databases, and the results are consistent. This suggests that AKR7A3 may serve as a potential biomarker for early diagnosis of HCC. Subsequent in vitro cell function tests also showed that overexpression of AKR7A3 significantly inhibited the proliferation, migration and invasion of HCC cells. Through AKR7A3 gene overexpression and second-generation transcriptome sequencing technology, differentially expressed genes were analyzed. KEGG pathway enrichment analysis showed that significantly affected genes may affect the PI3K signaling pathway, which can also regulate the expression of hypoxia-inducible factor 1α (HIF-1α). Therefore, the expression of HIF1α protein and VEGF protein, a downstream gene of HIF1α protein, was detected [32]. Western blot analysis verified that AKR7A3 overexpression was associated with changes in the HIF‑1α/VEGFA signaling pathway in Huh7 and SNU-387 cell lines.However, there was no significant difference in the expression of HIF-1α in Hep-G2 cell lines, It may be because that hep-G2 cell line is hepatoblastoma, so its development are different from HCC. Several articles have so far reported the anticancer effects of AKR7A3. In breast cancer, patients with high expression of AKR7A3 often have a longer survival period, simultaneously down regulate HIF-1α/ VEGFA signal pathway inhibits the activity of endothelial cells in peripheral blood vessels through paracrine pathway, thereby inhibiting tumor angiogenesis [7, 33]. An important step in the process of tumor migration and distant metastasis is through the EMT pathway. The study found that AKR7A3 can significantly reduce the half-life of β-catenin protein, inhibit Wnt pathway and increase Ecadherin protein, thus inhibiting the development of EMT [34]. Knockdown AKR7A3 of MKN45 gastric cancer cells can reduce the adhesion protein E-cadherin, thereby reducing the cell adhesion ability, and promote the migration, development of gastric cancer [35, 36]. In addition, studies have shown that AKR7A3 is a biomarker of early liver cancer, which is closely related to clinical staging and clinical prognosis [37]. In the experiment on the function of AKR7A3 gene in liver cells, it was confirmed that AKR7A3 has been reported to reduce ERK, c-Jun and NF-κB signaling pathways, which plays a role in inhibiting the carcinogenicity and chemical resistance of liver cancer [38]. Several limitations of this study should be acknowledged. First, the sample size was relatively modest for an exome-chip association study, and the association between rs1738023 and female HCC susceptibility requires validation in larger independent cohorts. Second, individual-level AFB1 exposure biomarkers were unavailable, and AFB1-DNA adducts were assessed using a semi-quantitative method. Third, the functional experiments were mainly based on AKR7A3 overexpression, while loss-of-function, rescue, and allele-specific validation studies were not performed. Finally, although the PI3K/HIF-1α/VEGFA pathway was implicated by transcriptomic and protein analyses, further mechanistic studies are required to confirm its role. Despite these limitations, our findings provide evidence supporting AKR7A3 as a potential susceptibility gene and tumor suppressor in female HCC.

## Conclusion

In conclusion, combined with our research findings, we can speculate that under the high exposure pressure environment of AFB1, the expression of AKR7A3 gene of rs1738023 genotype is low, and its detoxification ability to AFB1 is limited, leading to the accumulation of AFB1-DNA and protein adducts in cells, and eventually developing into HCC under long-term exposure pressure. At the same time, AKR7A3 is a significant biomarker of HCC, with low expression in HCC tissue. AKR7A3 may act via the HIF-1α/VEGFA signaling pathway to inhibit tumor initiation, progression, invasion, and metastasis.At the same time, it reduces the toxicity and side effects of AFB1 on normal cells.

## Supplementary Information

Below is the link to the electronic supplementary material.

Supplementary Material 1.

Supplementary Material 2.

## Data Availability

Data are available from the author upon reasonable requests.The datasets generated and/or analysed during the current study are available in the Genome Sequence Archive repository, https://ngdc.cncb.ac.cn/gsa.

## Abbreviations

HCC: Hepatocellular carcinoma
AKR7A3: Aldo-Keto Reductase Family 7 Member A3
GWAS: Genome-wide association study
AFB1: Aflatoxin B1
SNPs: Single nucleotide polymorphisms
DMEM: Dulbecco’s Modified Eagle Medium
FBS: Fetal bovine serum
GEO: Gene Expression Omnibus
CCK-8: Cell count Kit-8
OD: Optical density
NC: Negative control
DEGs: Different expressed genes
PCA: Principal component analysis
IBD: Identity-by-descent
eQTL: Expression quantitative trait locus
