# Supplementary material for: AKR7A3 rs1738023 association with susceptibility to female hepatocellular carcinoma and its role in AFB1 metabolism and tumor
Source: Discov Oncol. 2026 Aug 24;17:1318. doi: 10.1007/s12672-026-05757-9 (PMC13558527; doi:10.1007/s12672-026-05757-9)
Supplement: Supplementary file 1 [file 12672_2026_5757_MOESM2_ESM.docx]

Table S1 The primer sequence

| **Gene** | **forward** | **reverse** |
| --- | --- | --- |
| **AKR7A3** | 5'-ATGAGGACAAGAATGGGAAACA-3' | 5'-GCAATGCCCTCAAAGTGGT-3' |
| **GAPDH** | 5'-TGTCAAGCTCATTTCCTGGTATG-3' | 5'-TCTCTCTTCCTCTTGTGCTCTTG-3' |

Table S2 Analysis for genotypes of candidate SNPs

| SNPs | Group | | OR (95% CI) | *P* |
| --- | --- | --- | --- | --- |
|  | Control | Case |  |  |
| rs1738023 |  |  |  |  |
| Additive |  |  |  |  |
| CC | 186 | 90 | 1 | 0.002 |
| TC | 91 | 15 | 0.034(0.19–0.62) | 0.000449 |
| TT | 32 | 0 | / | 0.997701 |
| Dominant |  |  |  |  |
| CC | 186 | 90 | 1 | 0.000005 |
| TC + TT | 123 | 15 | 0.25(0.14–0.46) |  |
